# Asserting the visibility of social work in science

**DOI:** 10.3389/fsoc.2024.1411781

**Published:** 2024-07-31

**Authors:** Ariadna Munté-Pascual, María Virginia Matulič-Domandzič, Ane López de Aguileta, Emilia Aiello, Carmen Elboj, Diana Valero, Patricia Melgar

**Affiliations:** ^1^Social Work Training and Research Section, University of Barcelona, Barcelona, Spain; ^2^Department of Sociology, Autonomous University of Madrid, Madrid, Spain; ^3^Department of Psychology and Sociology, University of Zaragoza, Zaragoza, Spain; ^4^Department of Education, University of Girona, Girona, Spain

**Keywords:** co-creation, interdisciplinarity, scientific impact, social impact, social work

## Abstract

Scientific evidence has shown that Social Work has frequently been considered a second-level discipline in the traditional sexist hierarchy, because pioneers and most social workers are women. The twofold objective of this article is to analyze the dynamics that overcome this consideration and to put forward actions to go further in the near future. The factors that limit these actions and those that make them possible are studied. This article exposes the dynamics of the current transformation of Social Work, namely, the increase in the importance of social impact in social research, the increase in interdisciplinarity, and the impact of interdisciplinary research.

## 1 Introduction

Social Workers often have to answer the question “What is Social Work?” This happens in both lay and scientific forums. This reality has to do with the invisibilisation of a field of the social sciences that, even with a Nobel Prize winner among the women protagonists of its history, has often been considered a second-tier field in the traditional hierarchy of the sciences and subordinated as a field of application of disciplines such as psychology and sociology (Shaw et al., 2006). To address this issue, the present conceptual article focuses on unveiling the scientific and social impact of social work and how it is responding to the current priorities of both citizens and scientific community. To do so, the present paper analyses the priorities of current scientific disciplines, how Social Work research is embedded in them, and future challenges in order to enhance both the scientific and social impact of it.

This paper is based on the international definition of Global Social Work approved by the IFSW (International Federation of Social Workers) General Meeting and the IASSW (International Association of Schools of Social Work) [International Federation of Social Workers (IFSW) and International Association of Schools of Social Work (IASSW), 2014]:

“Social work is a practice-based profession and an academic discipline that promotes social change and development, social cohesion, and the empowerment and liberation of people. Principles of social justice, human rights, collective responsibility and respect for diversities are central to social work”.

In this line, Bywaters (2008) explains how Social Work has struggled to be recognized with the same status as other social sciences in the different countries where it is developed. Its unequal status is reflected in elements such as its position in national academic institutions or the evaluation and funding of research, which means that Social Work is less able to secure the necessary resources to continue building the discipline in some contexts (Matthieu et al., 2008; Corvo et al., 2011; Acker and McGinn, 2021). Likewise, the same author presents how during the elaboration of the strategy for Social Work research in higher education institutions in the UK in 2006, it was highlighted that scientific production is a key element in the promotion of a higher status of Social Work research, which is limited in quantity, quality, and resources concerning other social sciences such as sociology or policy.

Nevertheless, in the last decades, a shift in all sciences’ priorities has been done. The most important research programs, such as the one from the European Commission, have established two criteria for all sciences: social impact and co-creation. In the following pages, it will be argued why this change has put Social Work in an advanced position, potentially changing the mentioned dynamics.

This article analyses the current challenges faced by Social Work to achieve both scientific impact and social impact from research. Based on the aforementioned challenges, a twofold objective is proposed:

1. To present the challenges Social Work research has experienced and is experiencing regarding scientific impact.
2. To highlight three dynamics of transformation that are overcoming the consideration of Social Work as a second-rate discipline by increasing the social and scientific impact of its research.
3. To expose ways that will break the traditional theory/research-practice dichotomy by transforming Social Work in the short-term future. These ways include the consideration of the scientific impact and social impact as two dimensions that, beyond adding up, multiply its transforming effect, and allow to be completely open to coordination with colleagues from all disciplines.

## 2 Challenges of social work research

Social Work research should be useful for professional practice, responding to its needs, and expanding the knowledge base with research to guide practice and policy (Maynard et al., 2014). Thus, professional practice must take into account the evidence to provide individuals, groups, and communities with the most effective intervention (Fong and Pomeroy, 2011). However, there is in Social Work a gap between research and practice (Rubin, 2015; Palinkas et al., 2017) that can be understood when analyzing the relationship that, historically, Social Work has had with research. Some contributions (Rosero-Labbé, 2006; Lorente-Molina and Luxardo, 2018) show how Social Work, initially considered an applied science, has gone from depending on the scientific knowledge of the so-called “fundamental” disciplines to producing its own knowledge base.

Despite the important scientific contributions of Social Work that also contributed to the advancement of applied sociology (Residents of Hull House. A Social Settlement, 1895; Richmond and Half, 1913; Richmond, 1922), the yearning for social recognition and scientific credibility caused the initial knowledge production to be subject to the epistemological and methodological precepts of “core knowledge.” Thus, logical positivism was adopted as the research model for Social Work by the National Association of Social Workers (NASW) in 1949. This positivistic logic supported by a dichotomous theory-practice model was not exempt from criticism because it did not fit with the ethical and epistemological values of Social Work. It was a dehumanized form of knowledge production that, for many Social Workers, failed to respond to social-disciplinary needs by moving away from its own ethos in favor of a supposed greater scientificity. However, it was not until the 1960s that alternative approaches appeared with force, such as those derived from the reconceptualisation movement in Latin America (Muñoz-Arce and Rubilar-Donoso, 2021), which advocated connecting research with the emancipation processes of communities.

In the 1980s, other epistemological perspectives appeared along the lines of constructivism, hermeneutics, phenomenology and dialectical materialism that attempt to unite the logic of intervention with the logic of research, given the parallelism of the methodological process of intervention with that of research (De Robertis, 1981; De La Red, 1993). Currently, the positions described here coexist, although reflection on this topic constantly leads to progress in the field of research. It is the case of “Evidence-Based Research,” which was born to emulate core knowledge but distance itself from “Authority-Based Practice” by including the practical knowledge of professionals and users of Social Services (Gambrill, 2019).

However, the controversy continues as a misuse of this proposal has been detected with the consequent criticism for being an instrument of the bureaucracies in which research and professional practice are developed and not inclusive of the voices of professionals and users (Udo et al., 2019; Jacobsson and Meeuwisse, 2020). We see, therefore, that the debate on research, publication and use of evidence in Social Work continues to be open in the international scientific community today.

Despite the road ahead, progress has been made today with contributions such as those of Trevithick (2008), who argues that Social Work is used to face complex problems and a multiplicity of tasks in which, to understand what is happening and to know the best way to intervene, it is necessary to make use of different types of knowledge. His proposal is along the lines of creating new opportunities for critical reflection on the creation of knowledge and research that provides scientific answers to this complexity (Maynard et al., 2014).

The elements to understand the lack of scientific impact are found in the origin and development of the discipline. The research path in the history of Social Work causes that, nowadays a part of Social Workers (both in the professional and academic sphere) do not assume scientific impact as one of the priorities of the discipline in response to a rejection of the positivist epistemological tendencies (considered dehumanising) of the “fundamental disciplines” that prevailed at the beginning (Rosero-Labbé, 2006; Tilbury et al., 2021). Starting from the premise that Social Work is a discipline that works for the promotion of “social change and development, social cohesion, and the empowerment and liberation of people” [International Federation of Social Workers (IFSW) and International Association of Schools of Social Work (IASSW), 2014], it is understandable that most research in Social Work has been and is oriented toward the achievement of social impact, which is defined as the social improvements achieved through the implementation of the results of a research or project, also valuing those empirical investigations or theoretical developments that have allowed achieving such social impact (Soler Gallart, 2017; Reale et al., 2018).

In this way, it is evident how research is a key element for Social Work to be considered a first-tier discipline based on its scientific impact, understanding it as “the capacity of founding new schools of thought and influencing future research in the field” (Reale et al., 2018, p. 300). However, it must be clarified that research in Social Work is unequally distributed, with the absence of knowledge from the Global South (Roche and Flynn, 2020).

Scientific impact is “related to supporting the creation and diffusion of high-quality new knowledge, skills, technologies and solutions to global challenges” (Flecha et al., 2018, p. 5). Social Work can benefit from scientific evidence of social impact, that is, the subset of scientific evidence that has generated an improvement toward the achievement of societal goals (Flecha, 2022). In addition, Social Work is increasingly having scientific publications (Brekke, 2012; Rodriguez Otero and Facal Fondo, 2019; Munté Pascual et al., 2020) with a high scientific impact (Hodge et al., 2016), as will be further discussed in the next pages.

## 3 Dynamics of transformation of social work

As Shaw et al. (2006) argue, disciplines are not static entities, but the consequence of negotiations and decisions. As discussed in the previous point, throughout the history of Social Work, it has been the subject of debate as to whether it should be considered a first-order discipline or a second-order discipline that applies knowledge from other disciplines such as sociology or psychology (Shaw et al., 2006; Fong, 2012).

Currently, there are some authors who recognize Social Work as an academic discipline which bases its knowledge on three headings: practice/practical/personal knowledge, factual knowledge and theoretical knowledge (Trevithick, 2008). And it is within the latter where we can find theories from other disciplines that contribute to building the knowledge area of the Social Work aimed at understanding people, situations and events. Likewise, the intersection of the remaining knowledge identified by Pamela Trevithick becomes the field of knowledge itself that can nourish other disciplines on equal terms.

Social Work research is undergoing changes that contribute to overcoming the consideration of Social Work as a second-level discipline, increasing the social and scientific impact of Social Work research. In addition to the internal debates and dynamics of the discipline, there are trends of change in the social sciences in general.

Specifically, among the dynamics contributing to the current transformation of Social Work, we highlight: (1) the importance that is finally being given to the social impact of research within the social sciences, a dimension in which Social Work is gaining importance; (2) the greater openness to interdisciplinary and egalitarian work by researchers from different social sciences and health sciences who now treat their Social Work colleagues as equals and not as subordinates; and (3) the achievement of a scientific impact similar or superior to colleagues from other social sciences.

### 3.1 The requirement of social impact and co-creation

All sciences are experiencing a movement toward the social, political and scientific impact of their research, promoting a response to societal objectives. An example of this is how the impact of research has acquired greater importance in research policies: “a key concern of contemporary research policies is to demonstrate the ‘impact’ of research, or the value that public investment in research generates for increasing scientific competitiveness and excellence of the country, wealth creation, productivity, and social well-being” (Reale et al., 2018, p. 298). Increasingly, citizens, politicians and other organizations are demanding that science has a positive impact on the acquisition of democratically set goals, such as the Sustainable Development Goals (Flecha, 2022).

As previously mentioned, two priorities are being incorporated into all sciences, in the most relevant research programmes: social impact and co-creation (Flecha et al., 2018). Scientific literature has identified successful strategies for enhancing the scientific, political, and social impact of research projects (Aiello et al., 2021). Two of those strategies are especially relevant for Social Work in achieving the mentioned social impact: having, from the outset of the research, the objective of attaining social impact; and being meaningfully involved in co-creation with stakeholders throughout the research’s lifespan (Aiello et al., 2021).

These two elements are intrinsic to Social Work. Concerning the first strategy, social impact or the improvement of social situations is the ethos or *raison d’être* of the discipline itself. This fact is evidenced in the very definition of Social Work. In it, the promotion of “social change and development, social cohesion, and the empowerment and liberation of people” is stated as an objective. Therefore, Social Work research tends to seek social impact, i.e., improvement in some social domains.

To respond to the requirements of a greater social impact of research, there is a need for research that responds to the needs of professionals and the population, and specifically that contributes to the achievement of goals democratically set by society (Flecha et al., 2018). To achieve this goal, research as a tool to improve practice has been present since the beginning of Social Work. The pioneers of this discipline already dedicated their efforts to the professionalization of Social Work based on the application of science to practice. An example of this is the intense research work of Mary Richmond on which she was able to establish the bases of knowledge and intervention of Social Case Work (Richmond, 1922). Richmond, in her book Social Case Work published in 1922, already stated that casework Social Work was interrelated with social reform and social research (Richmond, 1995). The latter not only aims to generate a knowledge base but also to generate knowledge that is useful for use (Richmond, 1995). Similarly, Jane Addams, the most prominent figure of the Settlement House movement, understood that social problems transcended individual factors so the research about the Hull House was conducted to serve as a basis for social reform (Addams, 1910), all this being summarized in the maxim of the three Rs: Research, Reform and Residence (Miranda Aranda, 2004; Branco, 2016). It must be highlighted that the topics the founders of Social Work dealt with in their work and research are what now are the main priorities in the main research programs, such as: overcoming poverty, gender equality, quality education for all (especially the most vulnerable ones), etc.

When looking at the social impact seeked and achieved by these women pioneers of the discipline, the improvements of the lives of Chicago’s industrial areas stands out. The work by Jane Addams clearly exposes the climate of peace among very culturally and ethnically diverse populations; the increase of educational level and job expectations of the neighbors; the overcoming of loneliness among many of them, with a focus on the elderly; and the advocacy for children’s rights, having a great social impact not only in their community but also in the United Stated of America and globally through the child labor reform or the modern juvenile court system, among others (Addams, 1910).

The second strategy, co-creation, also places Social Work in an advanced position because its object of research is the subject of the intervention itself and in the intervention itself it gains access to practical knowledge that can be theorized in a rich feedback process in which the voice of the research subjects themselves is included in an egalitarian dialog. All this makes it easier to conduct transformation-oriented research that takes into account the voice and participation of the community and stakeholders throughout the research, especially the most vulnerable groups.

This increase in the importance of the social impact of research has also promoted the development of new methodologies in sciences that include the voices of the people under investigation, not as an objective in itself but as a way to overcome situations of inequality based on scientific rigor and the social usefulness of the research (Valls and Padrós, 2011). Furthermore, based on the critical reflection of the elements that perpetuate situations of inequality, as well as those that transform them, work is done to overcome inequalities and social exclusion, i.e., they pay special attention to the social impact during and after the research, such as the Communicative Methodology (Roca et al., 2022). This methodology was pioneer in the development of the previously mentioned criteria of co-creation and social impact.

In this line, research is being carried out that goes further, conducting studies that, from the transfer of the knowledge produced, improve the social reality genuinely, that is, research that generates Social Creation (Aiello and Joanpere, 2014). Specifically, the Communicative Methodology has been recognized by the European Commission as an approach to overcoming situations of exclusion of vulnerable groups. Not only does the inclusion of the participants of the research improve their outcomes; it improves the scientific outcomes of the research by avoiding wrong interpretations of reality, among others (Roca et al., 2022).

In this way, the Communicative Methodology allows for conducting research following the principles of Social Work, such as the struggle for human rights and social justice and cohesion [International Federation of Social Workers (IFSW) and International Association of Schools of Social Work (IASSW), 2001]. It must be stated, of course, that power relations are also present in the research practices of many scholars (Hancock, 1987); however, we argue that the origin of the discipline was not so and that it responds into these new priorities that are now a requirement of all sciences: social impact and co-creation.

### 3.2 Interdisciplinarity and its increase

Currently, interdisciplinary work is essential to respond to social needs. The incorporation of new perspectives generates novel combinations that enable innovation (Uzzi et al., 2013). This conception of research can provide new answers to complex problems, such as those with which Social Work works, for which approaches from a single discipline do not cover its globality (McCallin, 2006; Clarke et al., 2012).

Given the need for interdisciplinary research, Social Work is in a privileged position. This is because a great variety of the problems to which an interdisciplinary response must be given are social situations with which Social Work has historically worked (Nurius, 2017). That is, from Social Work, the object itself implies a need for interdisciplinarity. In his analysis of the object of Social Work, Zamanillo proposes “phenomena related to psychosocial distress” (Zamanillo Peral, 1999, p. 29) as the object of the discipline, dealing with everything that generates human suffering, whether of socio-structural genesis (poverty, moral, social or cultural deprivation, dependence, etc.) or provoked by personal experience.

In this way, the wide range of fields in which Social Work intervenes and their complexity is evident, a fact that makes interdisciplinary intervention essential. The specificity of intervening in the space of individual-society interaction allows Social Work to draw on knowledge from other disciplines and to have its knowledge that is also necessary for other fields of knowledge such as sociology or medicine. On the other hand, being present in a large number of institutions such as schools, hospitals, penitentiary centres, social services, etc. provides it with a long experience in working with other disciplines (Nurius and Kemp, 2012).

### 3.3 Scientific impact and its increase

In recent decades, the social sciences have made great strides in their scientific impact (Chaves-Montero and Vázquez-Aguado, 2021). However, although there are more and more Social Workers who, aware of the importance of scientific impact, orient their efforts in research and scientific publications, even today Social Work has a limited impact in the scientific field (Rodriguez Otero and Facal Fondo, 2019; Wu et al., 2022).

One of the elements that explain this fact is that some Social Work researchers have conducted very important social studies but reject or do not care about their publication and evaluation by the international scientific community (Tilbury et al., 2021).

Another element that explains this fact is the professionalizing tendency of the first schools of Social Work. Likewise, in certain social contexts, as in Spain, the consideration of Social Work as a professionalizing discipline has led to limitations in the advancement of research and its impact. Continuing with the Spanish case, the establishment of the European Higher Education Area (EHEA) has made it possible to put Social Work studies on an equal footing with other social sciences, opening the door to the emergence of master’s degrees, doctorates, and Social Work research groups (Martínez-Brawley and Vázquez Aguado, 2008).

Interdisciplinary work is making it possible not only to increase the social impact of research but also its scientific impact. In this way, Social Work is matching the impact of professionals from other sciences.

The increase of this impact in Social Work can be observed in elements such as the increase in the number of journals indexed on the Web of Science in Social Work or the increase in the scientific impact of some Social Work authors. In relation to Social Work journals ranked in the Journal Citation Reports (JCR), in the Social Sciences Citation Index, these have increased from 32 in 1997 to 44 journals in 2022, where their impact has increased from a Journal Impact Factor of 1,119 relative to the journal with the highest impact in 1997 to 6,116 in 2023. Likewise, interdisciplinarity is also present in scientific journals. Some JCR journals classified in Social Work are not considered disciplinary journals but some of the journals with the highest impact in the Journal Citation Reports are journals shared with disciplines such as psychology, psychiatry, or criminology and fields such as family studies.

Regarding the scientific impact of researchers in Social Work, Hodge et al. (2016) state in their study that there are researchers in Social Work who are achieving a high scientific impact. These same authors analyse the scientific impact of authors in Social Work from the analysis of the h-index and m-index. From their analysis, they show how in Social Work there are numerous authors with an h-index with values between 33 to 93 and an m-index with values between 1.13 and 3.33 (Hodge et al., 2016). Although, as the authors state, the scientific impact is not evidenced only from these indexes, they give us an orientation of the great scientific impact that authors in Social Work are achieving from scientific articles, both individually and by sharing authorship with other Social Work professionals or from other disciplines (Hodge et al., 2016).

Through these three dynamics, in recent years, Social Work is increasing its social and scientific impact. This is helping overcome the consideration of Social Work as a second-rate discipline, equaling other disciplines in the traditional hierarchy of sciences.

## 4 Pathways for further transformation of social work

The current changes in the conception of the social sciences and their impact open new avenues for further transformation of the discipline. Specifically, the consideration of the scientific impact and social impact as a multiplication and not as a sum and the total openness to coordination with colleagues from all disciplines, without accepting subordination, stand out.

About the first proposed pathway, the social and scientific impacts of research are not isolated from each other but feedback on each other. To begin with, when interventions that are based on scientific evidence are more effective, which is a strong argument for using knowledge from research in professional practice, thus increasing the social impact of research.

Thanks to the social impact of research, researchers have a large volume of evidence available for publication, allowing it to be evaluated and used by the scientific community, increasing its scientific impact. In addition, scientific impact allows greater dissemination of research results, so that knowledge and evidence reach more researchers and professionals. By transferring this evidence, the radius of the social impact of the research is expanded.

An example of this multiplying process is the case of the school of La Paz and the neighborhoods of La Estrella and La Milagrosa in Albacete, Spain. These neighborhoods were characterized by the high rate of poverty and marginality experienced by their inhabitants. From the *INCLUD-ED project. Strategies for inclusion and social cohesion in Europe from Education* of the European Commission’s Sixth Framework Program, scientific evidence-based actions were implemented in the school and neighborhood. In this community, an idea was not tested to respond to the needs of the community, but rather successful educational actions (SEAs) were implemented based on scientific evidence that had been shown to reduce inequality and social exclusion (Gatt et al., 2011). The implementation of SEAs made it possible to overcome the situations of inequality and social exclusion experienced by neighbors, breaking the circles of poverty that affected the families living in the neighborhood.

In order to clarify the nuances between scientific impact and social impact, the previous case will be used. The scientific evidence created through the mentioned project was disseminated in scientific journals, achieving a great number of citations and validation from the scientific community (that is, scientific impact). Later, this evidence was transferred to new contexts. However, this does not mean social impact, only transference; social impact only arrives with evidence of the improvements in the new contexts of the application of that scientific knowledge in relation to societal goals such as the SDGs (Flecha et al., 2018).

Finally, scientific impact allows the scientific community to use the knowledge generated to continue expanding the knowledge base of Social Work. In this way, Social Work researchers publish scientific articles in the best scientific journals and in turn, as mentioned above, new Social Work journals are created that compete at the same level as journals from other disciplines. All this makes it possible to increase the visibility of Social Work as a discipline and therefore contributes to overcoming the consideration of Social Work as a second-tier discipline, generating interest in Social Work from other disciplines and therefore enabling new opportunities for collaboration with other disciplines.

Currently, there is a need for interdisciplinary research that goes beyond the social, natural or health domains, intending to inform policymakers in a comprehensive manner (Holm et al., 2013). This brings us to the second proposed pathway; to be fully open to coordination with colleagues from all disciplines.

From Social Work, this openness is done without accepting subordination, and without abandoning the knowledge and expertise of Social Work, but rather defending the knowledge that is proper to the discipline and profession (Golightley and Holloway, 2017). Likewise, this collaboration takes into account that the more open the collaboration, the better the results for science and society, allowing us to respond to changing and complex needs that cannot be addressed from a single field of knowledge, but that “increasingly require a mix of knowledge and skills transcending any one discipline” (Nurius and Kemp, 2012, p. 550). In interdisciplinary research, each discipline brings its knowledge and practice to the dialog with the other disciplines (Lach, 2014). Overcoming the subordination of Social Work in these dialogs involves overcoming its conception as a second-level discipline. By participating in interdisciplinary research without subordination, it will be possible to respond to previously unmet needs, allowing researchers “…to understand the wider impact of their research and their “home” discipline while also contributing to wider societal questions” (Bridle et al., 2013, p. 23), as well as to ask themselves more innovative and impactful research questions (Bridle et al., 2013). Interdisciplinarity allows for the creation of new and useful knowledge from the combination of knowledge (Wang, 2016). Thus, from interdisciplinary work, not only the scientific impact but also the social impact of their research is increased.

This focus on social impact poses Social Work in an advanced position in the current scientific revolution toward these priorities. A clear example is the case of the great social impact achieved through co-creation with Roma women, helping in the overcoming of stereotypes and barriers these population faced in very diverse fields such as education, social services or healthcare system (Valero et al., 2020; Samyn et al., 2024). Some examples in the cited works include the Roma women’s student gatherings, that have had a great social impact in the access of Roma women to quality education (SDG 4).

In addition, the knowledge created in this topic through Social Work has been used to address other needs of Roma women such as antenatal care or dialogic leadership in the educational field (Khaqan and Redondo-Sama, 2023; Claisse et al., 2024) with social impact. These mentioned works have cited and used scientific knowledge from Social Work in their own fields; this is only an example of the advanced position of Social Work in these new priorities. It is argued, therefore, that this fact makes the discipline specially prepared to offer its knowledge to other disciplines who are newly incorporating the criteria of social impact and co-creation in their knowledge creation.

## 5 Discussion and conclusions: implications for social work

Throughout history, Social Work has been considered a second-rate discipline. Faced with this situation, Social Workers have not stood still, but have worked to overcome this consideration. In this process, the social and scientific impact of research is not only a key element to promoting a higher status of Social Work at the scientific level, but also to increase the impact and effectiveness of interventions. Social Work “promotes social change and development, social cohesion, and the empowerment and liberation of people” [International Federation of Social Workers (IFSW) and International Association of Schools of Social Work (IASSW), 2014], so developing rigorous research oriented to social and scientific impact has the ultimate goal of achieving a solid body of knowledge that allows us to overcome the gap between research and practice that still exists today.

To achieve this objective, there are dynamics in social sciences research in which Social Work is in an advanced situation. This discipline is especially prepared to face the challenges of current global scientific research in its priorities of social impact and co-creation. Regarding social impact, its proximity to social situations is not a problem but an advantage for achieving what is now considered objectivity and scientific impact. This allows Social Work to have an almost immediate repercussion on social impact, which enhances its possibility of improving its scientific status. Previous evidence has shown that an orientation from the beginning toward achieving social impact is in fact key to achieve it (Aiello et al., 2021). In addition, due to its long history of collaboration with other disciplines, for example in the field of health, Social Work is also in a privileged position for interdisciplinary work, which is essential to address current needs that cannot be met by a single discipline and are the driving force for greater impact, not only socially but also scientifically.

In regard with co-creation, from the origins of pioneers like Jane Addams, it can be seen that the actions taken at the Hull House she co-led did not have a top-down design where people with university degrees decided what was best for that new neighborhood they had gone to live in. They dialogued on an equal footing with people in poverty or illiteracy, including victims of violence or labor exploitation. The objectives and actions of the Hull House were co-created with all the people involved and, in Addams’ own words, it can be seen how she also did not accept ideological impositions from other people with power who wanted to decide what the inhabitants and members of that movement would do. Thus, the work in co-creation from the beginning of the project marked both what was relevant to address and how it would be done. If we turn to the first writings in which Social Work is built, we can see how Jane Addams dialogued and co-created with the most diverse neighbors in an egalitarian dialog as a forms of knowledge creation:

“I addressed as many mothers' meetings and clubs among working women as I could (…) I am happy to remember that I never met with lack of understanding among the hard-working widows (…) There was always a willingness, even among the poorest women, to keep on with the hard night scrubbing or the long days of washing for the children's sake” (Addams, 1910, p. 205).

Even though the current dynamics of transformation are increasing the social and scientific impact of the discipline, there are still avenues for greater consideration of Social Work as a discipline of the first order. Two ways stand out, the consideration of social and scientific impact as a multiplication and not as a sum, i.e., the feedback of social and scientific impacts. By transferring this evidence, the social impact of research is greater. Thanks to this social impact, researchers have a large volume of evidence to publish, allowing it to be evaluated and used by the scientific community, thus increasing scientific impact. Finally, the second way that stands out is to open completely to collaboration with other disciplines. With strong Social Work, it is possible to open to this collaboration without accepting subordination, but by placing itself in a position of equality.

## 6 Conclusion

The current scientific revolution that is requiring the criteria of social impact and co-creation for all sciences is changing the visibility and status of Social Work. Although it has long been considered a second-level discipline and many of the social workers (most of them women) have been invisibilised, Social Work is now in an advanced position. The focus of social impact and co-creation has been in the foundations of the discipline, in both research and practice; this is why it is argued that Social Work is specially prepared to work and help other areas of knowledge to fulfill the now required criteria. In addition, the long tradition for interdisciplinary work that has also been evidenced in the previous lines is increasingly being valued by the most important research programmes such as that of the European Commission.

## Author contributions

AM-P: Conceptualization, Formal analysis, Funding acquisition, Investigation, Supervision, Writing – original draft. MVM-D: Formal analysis, Investigation, Writing – review & editing. ALA: Formal Analysis, Investigation, Writing – original draft. EA: Formal analysis, Validation, Visualization, Writing – review & editing. CE: Validation, Visualization, Writing – review & editing. DV: Validation, Visualization, Writing – review & editing. PM: Validation, Visualization, Writing – original draft.
